# Pathophysiological changes and injury markers for acute lung injury from blunt impact in infant rabbits

**DOI:** 10.3389/fped.2024.1354531

**Published:** 2024-06-07

**Authors:** Ke Wang, ZhenPeng Huang, JiaWei He, LingWang Kong, Mingwei Chen

**Affiliations:** ^1^ Department of Respiratory and Critical Care Medicine, The First Affiliated Hospital of Xi'an Jiaotong University, Xi'an, Shaanxi Province, China; ^2^ The Clinical Medicine Department, Xi'an Medical University, Xi'an, Shaanxi Province, China; ^3^ Faculty of Nursing, Guangxi University of Chinese Medicine, Nanning, Guangxi, China

**Keywords:** blunt impact lung injury, acute lung injury, circulatory injury markers, infant rabbits, inflammatory response

## Abstract

**Background:**

Traffic accidents, particularly blunt impacts, cause serious injuries in children. We aimed to assess inflammatory and injury responses in infant rabbits subjected to acute lung injury resulting from blunt impact, with the goal of identifying potential circulatory injury markers.

**Methods:**

Forty 4-week-old infant rabbits were subjected to a right chest impact using a Hopkinson bar with 2,600 g. Computed tomography was employed to assess injury severity. Pathological changes were observed using hematoxylin and eosin staining in the control, 0, 24, and 72 h groups, post-injury. Immunohistochemistry was used to examine surfactant protein A (SP-A) changes in right lung tissues and upper main bronchi. Serum levels of interleukin-6 (IL-6), IL-8, and SP-A were measured using ELISA within 24 h post-injury in the control, 0 h, and 24 h groups.

**Results:**

Following blunt injury, significant increases were observed in blood white blood cell count (*F* = 101.556, *P* < 0.01) and neutrophil percentage (*F* = 104.228, *P* < 0.01), which gradually decreased after 24 and 72 h. The lung wet/dry weight ratio indicated significant edema (*F* = 79.677, *P* < 0.01), corroborated by hematoxylin and eosin staining showing edema, exudation, and marked granulocyte infiltration in the control, 0 h, 24 h and 72 h groups. SP-A levels decreased rapidly at 0 h, and recovered between 24 and 72 h in the right lung tissues (*F* = 6.7, *P* < 0.05), left lung (*F* = 15.825, *P* < 0.05) and upper main bronchi (*F* = 59.552, *P* < 0.01). The ELISA results showed increasing trends for the control and 0 h groups, while decreasing trends were observed in 24 h group for IL-6 (*F* = 58.328, *P* < 0.01) and IL-8 (*F* = 41.802, *P* < 0.01). Conversely, SP-A exhibited a decreasing trend in the control and 0 h groups but increased in the serum of 24 h group (*F* = 52.629, *P* < 0.01).

**Discussion:**

In cases of direct chest trauma in infant rabbits, particularly mild injuries without rib fractures. SP-A levels correlated with pathological changes across all groups and may serve as biomarkers for pediatric blunt lung impact.

## 1 Introduction

Road traffic injury presents a serious hazard to children, with traffic accidents accounting for 78% of severe injuries in this demographic, compared to 63% in adults (1, 2). The distinction in seating postures between children and adults in motor vehicles contributes to the increased incidence of chest and abdominal injuries in children, reaching as high as 13%, surpassed only by head and neck injuries (3). These injuries demonstrate notably high mortality rates (4). However, the clinical manifestation of lung injuries resulting from blunt impact is often atypical and progresses rapidly, posing a risk for misdiagnosis in clinical settings (5, 6).

Current research in automotive road safety includes studies utilizing dummies and child computed tomography (CT) finite element models to study the mechanical changes in the thorax post-injury (7, 8). The complexity of the thoracic structure, composed of various tissues and organs (such as ribs, heart, and lungs), poses challenges in detailed finite element modelling (9). Moreover, computer simulations fail to replicate the hemodynamic changes observed in animal experiments, which are essential for enhancing clinical diagnosis and predicting pathophysiological alterations (10).

Researchers have conducted impact experiments on young piglets to investigate the pathophysiological responses analogous to those observed in children during impact events (11). These studies predominantly focus on thoracic deformation and alterations in respiratory vital signs following impact at varying mechanical accelerations. However, they lack a comprehensive examination of post-injury changes and the mechanisms of repair (10). At the cellular level, investigations into the impact-induced secretion changes in alveolar type II epithelial cells (12) have focused on the alveolar surfactant production and cellular repair processes. Nevertheless, there is a lack of research on the broader *in vivo* pathophysiological dynamics.

In previous studies, it has been shown that patients with pulmonary contusions suffered from alveolar and capillary damage and inflammatory immune activation (13), which could contribute to an enhanced risk of acute respiratory distress syndrome (ARDS) (14). The chest bones of children are more elastic, which means that stresses are transmitted to the lung tissue more easily, and there are no significant external injuries following blunt chest trauma (2). This study aimed to investigate the inflammatory responses and injury alterations in infant rabbits, in the presence of radiographic minor injury, following acute lung injury to identify indicative circulatory markers (15).

## 2 Materials and methods

### 2.1 Animal experiment

Forty 4-week-old young rabbits (as infant for average human weaning stage for 6 months) (1) weighing between 0.9–1.0 kg were randomly assigned into four groups: control, 0-h (h), 24 h, and 72 h (each group of rabbits was assessed only at the corresponding time after injury). Animals of both sexes were randomly grouped. The rabbits underwent anesthesia with 3% pentobarbital administered intravenously. Post-anesthesia, the right thorax was impacted using a Hopkinson bar with 2,600 g force, focusing on the lower right thorax to avoid direct impact on the heart. The animal experiment was approved by The Medical Research Ethics Committee of the Xi'an Medical University and adhered to ethical experimental practices in rabbits. CT scans were performed by a veterinarian from the Jinghe Veterinary Hospital to categorize the control and injury groups (16, 17).

### 2.2 Physiological and tissue assessment

Blood pressure and oxygen levels were measured before and after the impact. Pathophysiological changes were observed. Routine blood examinations were conducted in each group to assess white blood cell (WBC) count and neutrophil percentage in the control, 0 h, 24 h, and 72 h groups. A 1 cm^2^ sample of right upper lung tissue was excised for wet weight measurement, followed by drying in an oven at 80 °C for 72 h. After reaching a constant weight, the dry weight was registered, and the wet/dry weight ratio was calculated to assess edema.

### 2.3 HE staining and pathological injury score

The right lung was embedded in a wax block and fixed with 5% paraformaldehyde solution for paraffin sectioning. Sections were stained with hematoxylin and eosin (HE) and examined pathologically under a light microscope.

Lung injury scoring criteria included four indices: alveolar congestion, infiltration, or aggregation of neutrophils in the alveolar cavity or vascular wall, epithelial exfoliation in the lumen, and thickened alveolar walls or hyaline membranes in the lining alveoli. Each index was scored as follows (18): 0 for no or very slight lesions, 1 for light lesions, 2 for medium lesions, 3 for severe lesions, and 4 for extremely severe lesions. The total score was used to assess acute lung injury severity. Two respiratory experts independently rated each sample, and the scores were then averaged.

### 2.4 Immunohistochemistry

The right lower lung, left lung tissues and the upper part of the main bronchus were fixed in 4% paraformaldehyde solution and subsequently paraffin-embedded. Tissue sections of 4-µm thickness were prepared and incubated with a surfactant protein A (SP-A) antibody (1:100; BA1730, Boster, China) overnight at 4 °C. This was followed by incubation with a secondary antibody conjugated to horseradish peroxidase. Both HE and immunohistochemistry (IHC) stained sections were examined under a light microscope (Olympus BX53, Tokyo, Japan).

### 2.5 ELISA

The levels of interleukin (IL) and SP-A_1_ in circulating blood were determined using enzyme-linked immunosorbent assay (ELISA) kits (CSB-E06903Rb for IL-6, CSB-E06905Rb for IL-8, and CSB-EL021168RB for SPA; Cusabio, China). Serum samples from the control, 0 h, and 24 h groups were analyzed to investigate the changes within 24 h.

### 2.6 Statistical analysis

Statistical analysis was performed using SPSS version 23.0. Data for continuous variables were presented as means ± standard deviation. The analysis included variance analysis and single-sample *t*-tests. Graphs were generated using Origin 2024. Statistical significance was indicated by *P* < 0.05.

## 3 Results

### 3.1 Injury assessment and physiological responses

**Figure 1:**
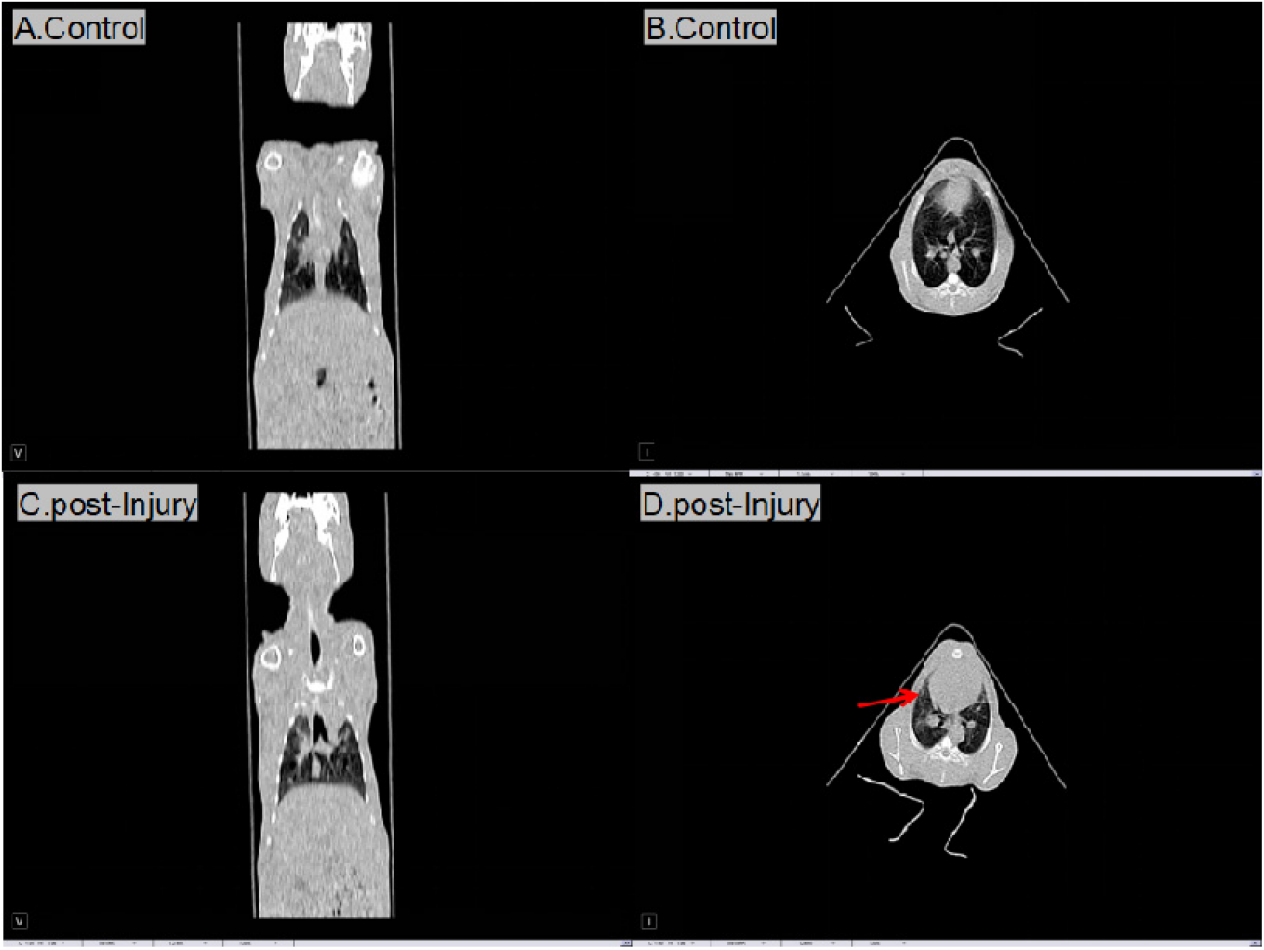
Computed tomography imaging of control and injury groups.

Control group and the right lung in the impact injury were assessed immediately (0 h group) to clarify the condition of the injury in CT. Following injury, effusion and a dense shadow were observed in the right lung (Figure 1). Additionally, no rib fractures were observed in any of the animals.

**Figure 2:**
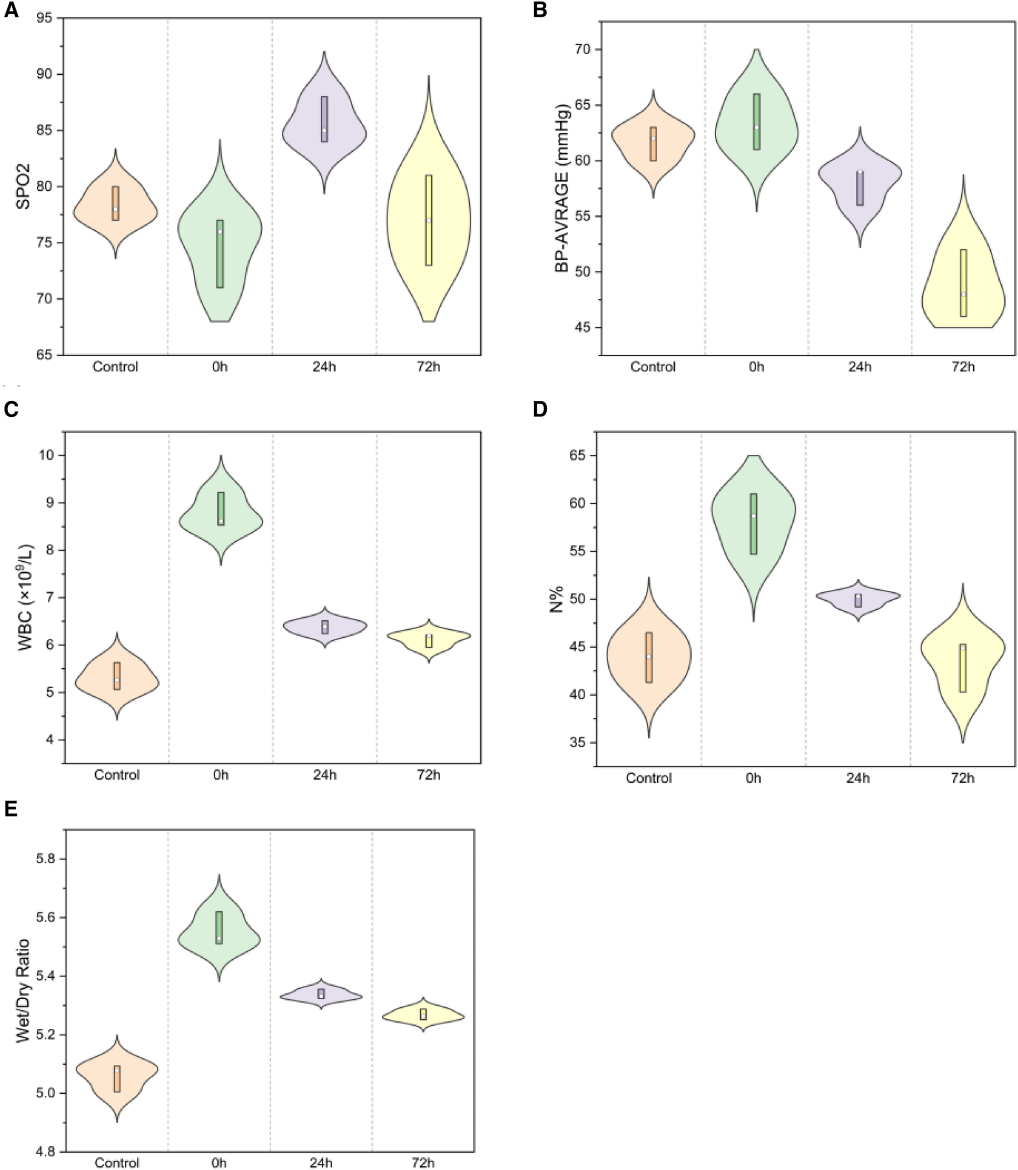
Spo2 and average blood pressure for (**A,B**) [(**A**) SpO2: *F* = 8.199, *P* < 0.01; (**B**) Average blood pressure: *F* = 24.566, *P* < 0.01]; WBC and neutrophil percentage for (**C,D**) [(**C**) WBC *F* = 101.556, *P* < 0.01; (**D**) neutrophil percentage *F* = 104.228, *P* < 0.01]; Lung wet/dry weight ratio (**E**) (W/D, *F* = 79.677, *P* < 0.01).

SpO_2_ and average blood pressure demonstrated significant differences across the control, 0 h, 24 h, and 72 h groups (Figure 2A,B). Compared to the control group, post-injury, SpO_2_ exhibited a decrease at 0 h, peaked at 24 h, but subsequently decreased at 72 h (78.33 ± 1.53 in the control group, 74.67 ± 3.21 in the 0 h group, 85.67 ± 2.08 at 24 h, and 77.00 ± 4.00 at 72 h; *F* = 8.199, *P* < 0.01). Compared to that in the control group, the average blood pressure increased in 0 h and then decreased (61.67 ± 1.53, 63.33 ± 2.52, 58.00 ± 1.73, 48.67 ± 3.06; *F* = 24.566, *P* < 0.01). Post-injury, WBC count increased rapidly and then gradually decreased after 24 and 72 h (Figure 2C, 5.32 ± 0.29, 8.79 ± 0.38, 6.38 ± 0.14, 6.12 ± 0.15; *F* = 101.556, *P* < 0.01), with a similar trend for *N* % (Figure 2D, 2.37 ± 0.08, 5.45 ± 0.29, 3.19 ± 0.11, 2.55 ± 0.36; *F* = 104.228, *P* < 0.01).

The lung wet/dry weight ratio indicated significant edema immediately post-injury, which gradually diminished at 24 h and markedly decreased at 72 h (Figure 2E, 5.06 ± 0.05, 5.55 ± 0.06, 5.34 ± 0.02, 5.27 ± 0.02; *F* = 79.677, *P* < 0.01).

### 3.2 HE staining

**Figure 3:**
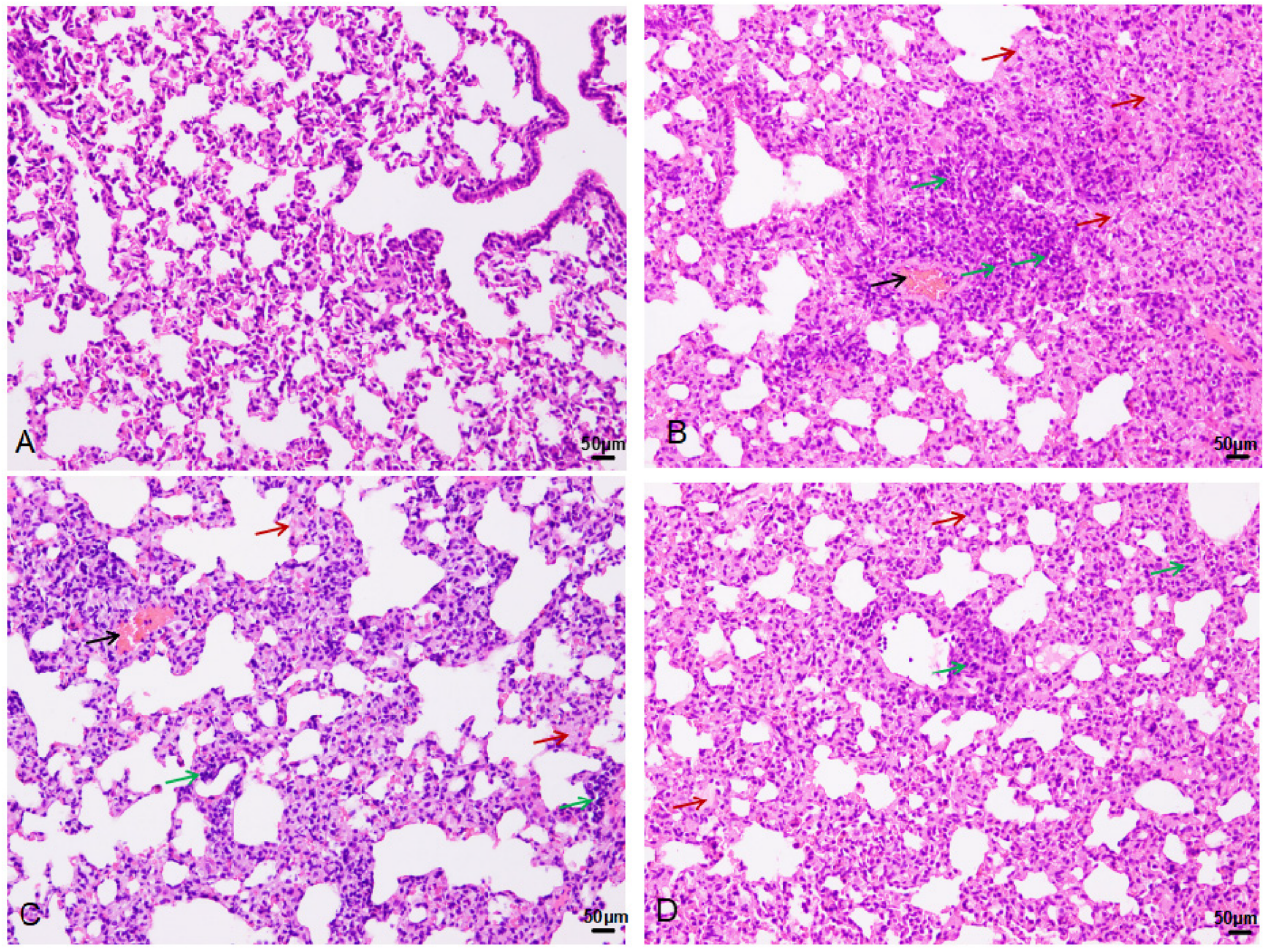
HE staining in the control (**A**), 0 h (**B**), 24 h (**C**), and 72 h (**D**) groups (200×): black arrow-bleeding, red-exudation, green-inflammatory cells infiltration.

**Figure 4:**
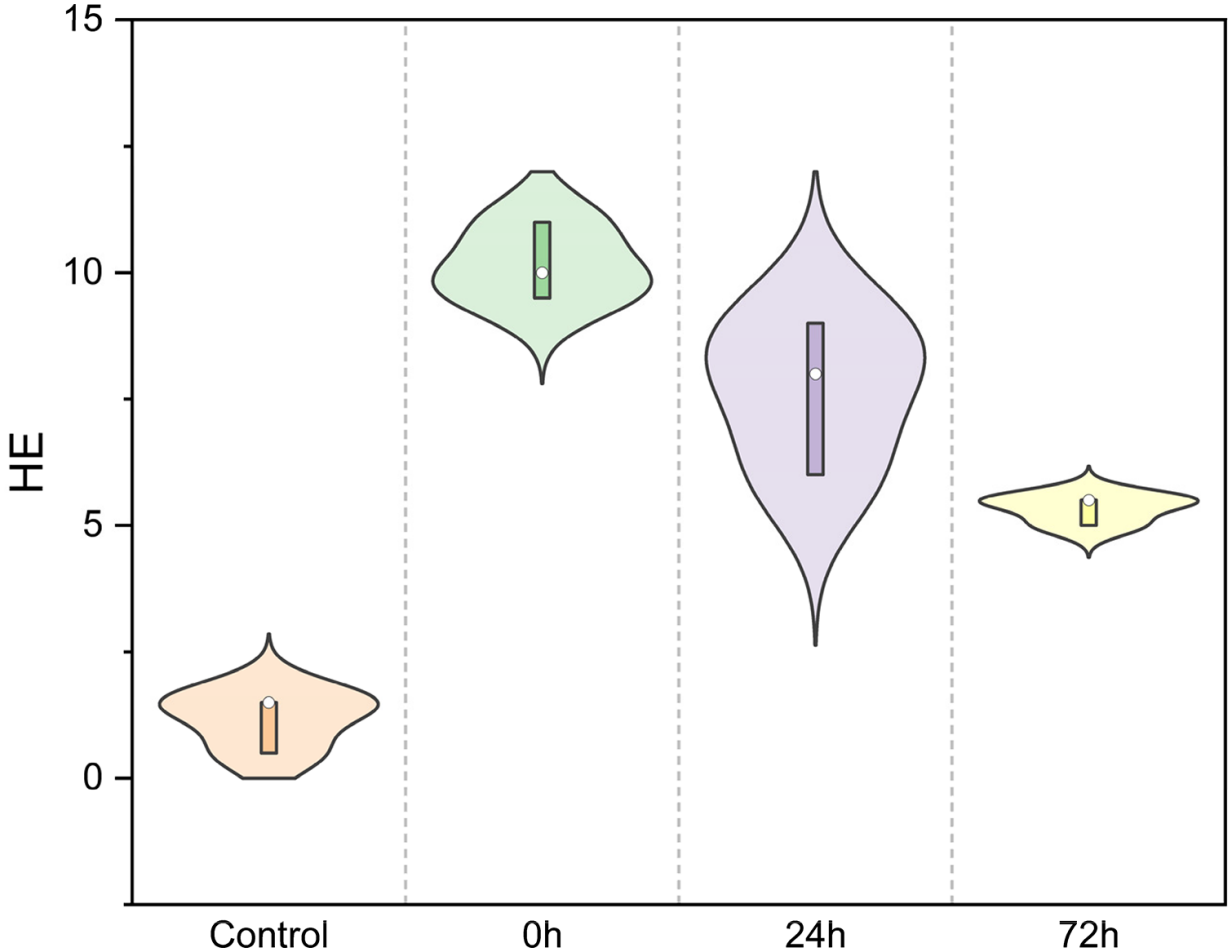
HE staining scores in the control, 0 h, 24 h, and 72 h groups (*F* = 52.7, *P* < 0.01).

Pathological examination of lung tissue revealed extensive interstitial and alveolar hemorrhage, rupture, and fragmentation of alveolar walls. Edema, exudation, and substantial granulocyte infiltration were observed in the pulmonary interstitial tissue and alveolar cavities. There were instances of erythrostasis and leukocytic margination in the alveolar capillaries, along with local pulmonary atelectasis (Figure 3). Moreover, cilia lodging and rupture of airway smooth muscle fibers were observed in the upper main bronchus. Lung injury scores (Figure 4) were 1.17 ± 0.58, 10.17 ± 0.76, 7.67 ± 1.53, and 5.33 ± 0.29 in the control, 0 h, 24 h, and 72 h groups, respectively (*F* = 52.7, *P* < 0.01, Figure 4).

### 3.3 Immunohistochemistry

#### 3.3.1 Right lung tissues for SP-A

**Figure 5:**
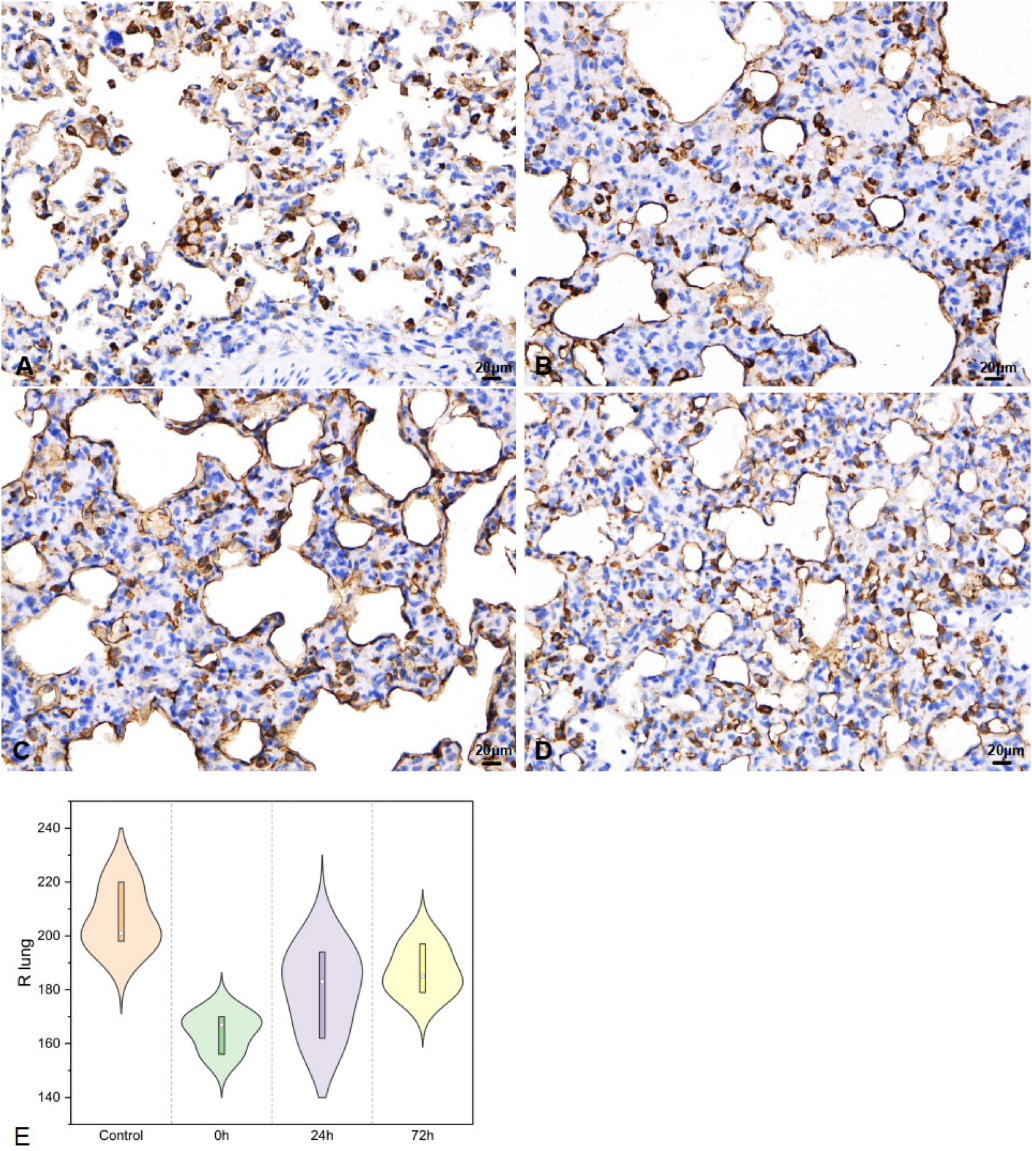
SP-A in right lung tissues IHC (400×; *F* = 6.7, *P* < 0.05). (**A**–**D**) were control/ 0 h/ 24 h/ 72 h group respectively. (**E**) was SP-A IHC changes in right lung tissues.

In the right lung tissues, SP-A secretion (Figure 5) was 206.33 ± 11.93, 164.33 ± 7.37, 179.67 ± 16.26, and 187.00 ± 9.17 in the control, 0 h, 24 h, and 72 h groups, respectively (*F* = 6.7, *P* < 0.05).

#### 3.3.2 Left lung tissues for SP-A

**Figure 6:**
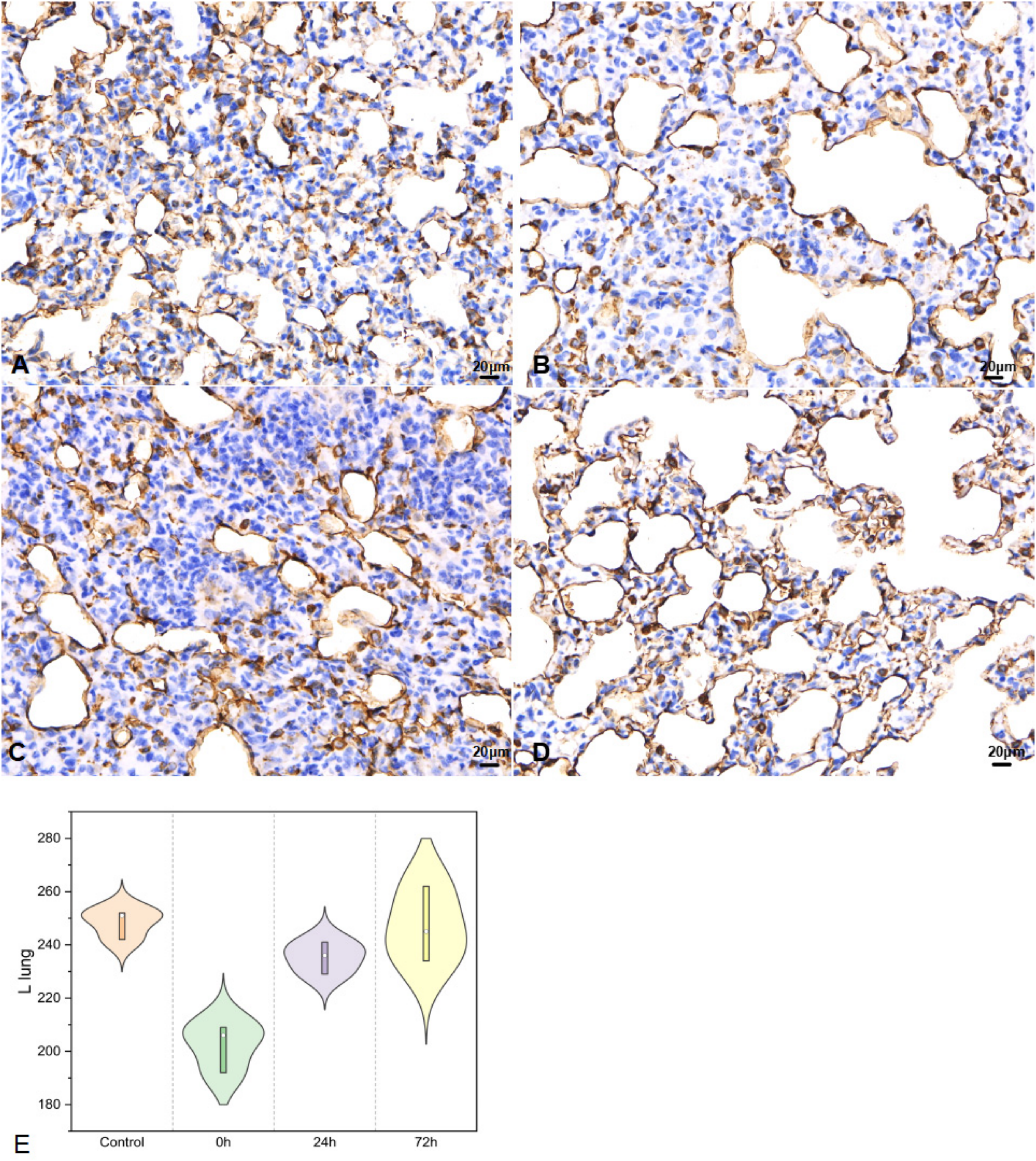
SP-A in left lung tissues IHC (400×; *F* = 15.825, *P* < 0.05). (**A**–**D**) were control/ 0 h/ 24 h/ 72 h group respectively. (**E**) was SP-A IHC changes in left lung tissues.

In left lung tissues, SP-A secretion (Figure 6) was 248.33 ± 5.51 in control, 202.33 ± 9.07 in 0 h, 235.33 ± 6.03 for 24 h, 247.00 ± 14.11 as 72 h group (*F* = 15.825, *P* < 0.05).

#### 3.3.3 Upper part of main bronchus for SP-A

**Figure 7:**
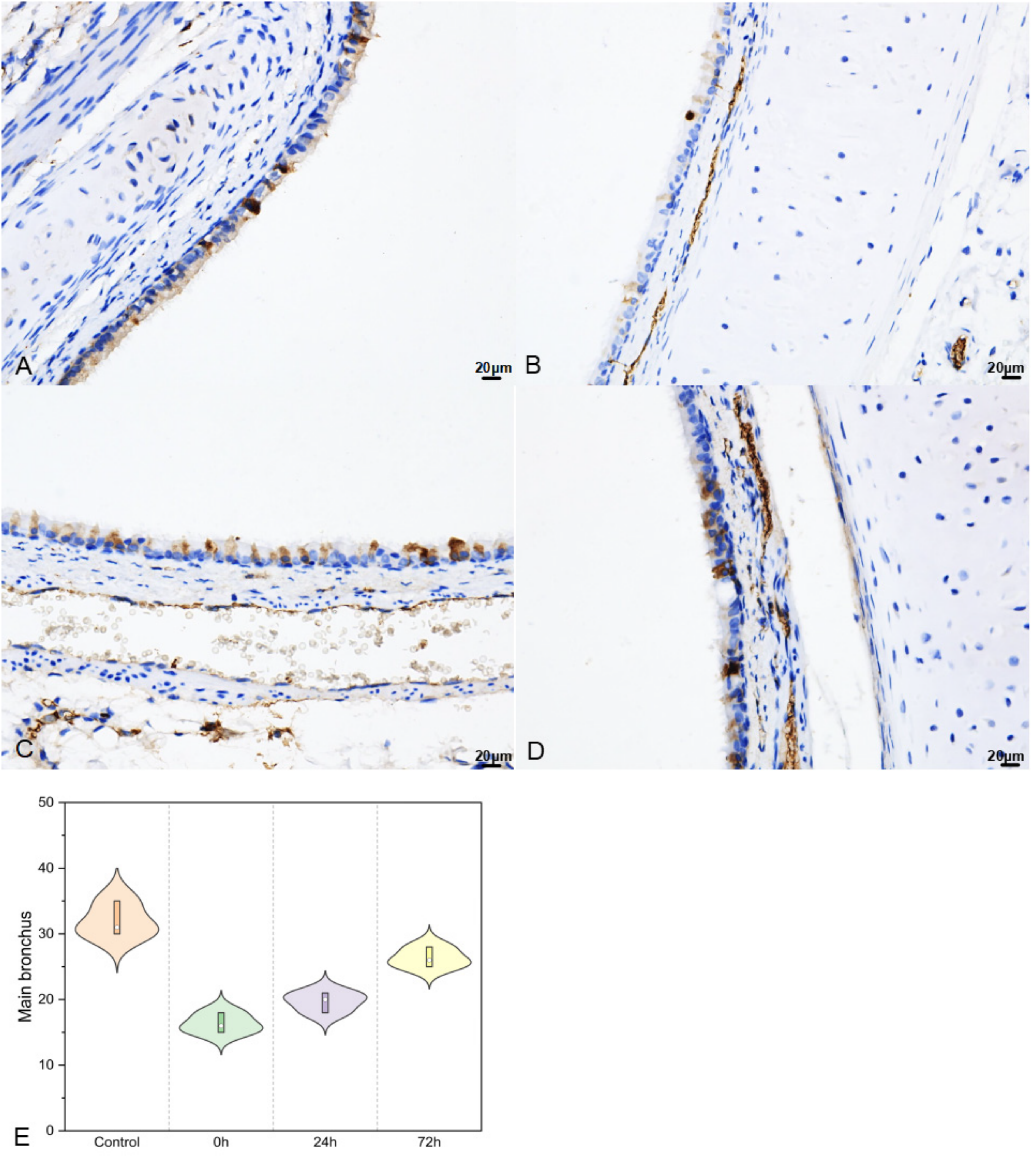
SP–A in upper part of main bronchus IHC (400×; *F* = 59.552, *P* < 0.01). (**A**–**D**) were control/ 0 h/ 24 h/ 72 h group respectively. (**E**) was SP–A IHC changes in upper part of main bronchus IHC.

Post-impact, in the upper part of the main bronchus, SP-A secretion (Figure 7) decreased in 0 h and then increased in 24 h and 72 h (32.00 ± 2.65, 16.33 ± 1.53, 19.67 ± 1.53, and 26.33 ± 1.53 in the control, 0 h, 24 h, and 72 h groups, respectively; *F* = 59.552, *P* < 0.01).

### 3.4 ELISA

**Figure 8:**
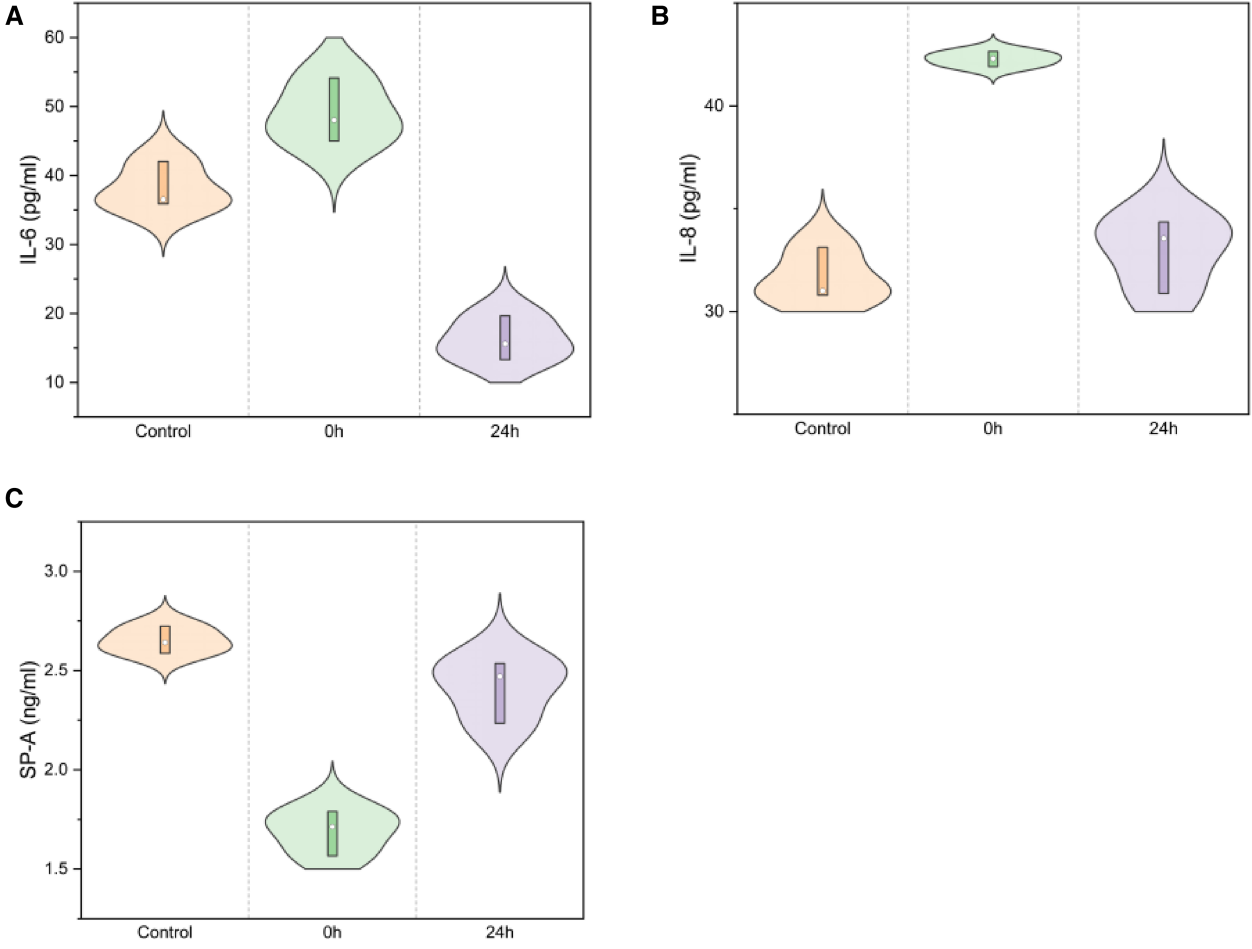
IL-6/IL-8/SP-A in serum ELISA for normal/0 h/24 h groups [(**A**) IL-6: *F* = 58.328, *P <* 0.01; (**B**) IL-8: *F*  = 41.802, *P <* 0.01; (**C**) SP-A: *F* = 52.629, *P <* 0.01).

SP-A levels decreased immediately post-injury and gradually increased in the 24 h group (2.65 ± 0.07, 1.69 ± 0.11, 2.41 ± 0.16; *F* = 52.629, *P* < 0.01), as illustrated in Figure 8 a. IL-6 levels rapidly increased at 24 h in infant rabbits (38.17 ± 3.36, 49.06 ± 4.60, 16.20 ± 3.22; *F* = 58.328, *P* < 0.01), similar to the trend observed for IL-8 (31.64 ± 1.29, 42.29 ± 0.37, 32.93 ± 1.82; *F* = 41.802, *P* < 0.01, Figure 8).

## 4 Discussion

Blunt chest trauma in children often goes undiagnosed or misdiagnosed (19). Lung contusions resulting from traffic accidents, explosions, or falls from heights account for 36% of pediatric injuries. The mechanical characteristics of the thorax in children differ from those in adults (11). Some researchers have isolated bone, lung tissue, muscle, and skin to measure their mechanical properties under compression and stretching, aiming to explore the mechanical distribution characteristics following lung injury (20, 21). However, these studies could not replicate the pathophysiological changes across different ages post-biological injury.

This study investigated the changes in lung injury in infant rabbits subjected to blunt impact force. Post-thoracotomy, no rib fractures were observed in any of the animals. Under light microscopy, HE-stained lung tissue exhibited alveolar wall destruction, along with secretion and neutrophil infiltration in the alveolar cavity. These changes initiated immediately following lung tissue injury. Differ from Wang H's research (22), the injury score peaked at 0 h. The findings suggest that acute lung injury can progress post-impact even in the absence of rib fractures and atypical clinical symptoms (6).

The lung wet/dry weight ratio indicated that the moisture content in lung tissue peaked immediately post-injury and then decreased at 24 and 72 h. Accompanied by a rapid increase in average blood pressure post-injury, SpO_2_ significantly decreased, recovered to a peak at 24 h, and gradually returned to normal by 72 h, unlike AoY's 6–12 h upto 80% of normal (23). This suggests that caution should be maintained beyond 24 h post-minor lung injury, with close monitoring up to 72 h. Routine blood examinations showed a rapid increase in WBC and neutrophils, which gradually normalized within 24 h and decreased by 72 h, inconsistent with 12–24 h increasing in Tong C's study (24).

Inflammatory factors play a crucial role in the transition of acute lung injury to acute respiratory distress syndrome (25, 26). To understand the changes in inflammation during the immediate inflammatory response within 24 h, we monitored the control group and the injury groups at 0 and 24 h using serological ELISA. Serum levels of IL-6 and IL-8 increased rapidly post-injury and then decreased at 24 h (27). Some researchers found that the levels of proinflammatory factors such as IL-6, and IL-8 were significantly increased in BALF and serum of ALI/ARDS children. IL-6 may be a pro-inflammatory factor in ALI and a promoter of the release of other inflammatory mediators. Early in ALI inflammation, IL-8 can promote neutrophil aggregation in the inflammatory lesion. In blunt infant-rabbit, it indicated certain clinical value for predicting the severity of ALI/ARDS and the treatment effect by monitoring serum changes in IL-6 and IL-8 (28, 29).

Predominantly synthesised and secreted by alveolar epithelial cells II, SP-A is the most abundant hydrophilic glycoprotein in the Surfactant protein, which should be an important role in immune defense and lung inflammation (30, 31). The serum levels of SP-A were significantly lower in the children with acute lung injury than in the healthy children. Serum ELISA demonstrated that SP-A, a potential marker of circulatory damage, decreased rapidly and then gradually recovered after 24 h.

The variation in SP-A levels is indicative of alveolar injury and repair (32). Regarding immature infant lungs, IHC of the right lung revealed a significant decrease in SP-A following acute impact injury, with gradual recovery observed between 24 and 72 h. Interestingly, we found that the SP-A changes in the left lung were similar to those seen in the injured lung. Moreover, in regions not directly impacted, bronchial SP-A changes mirrored those in lung tissue, showing a significant decrease immediately after injury and recovery within 24–72 h. Previous studies have revealed that post lung injury SP-A decreases (33), accompanying alveolar injury and secretion function, recruitment of neutrophils, and activation of macrophages that release a high number of inflammatory factors (34). This cascade exacerbates acute lung injury. Then, activated the hypothalamic-pituitary-adrenal axis and sympathetic nervous system alike (35), lung damage-repair balances were concomitant with inflammation in indirect part of lung. SP-A was a good indicator in ALI for babies.

In the course of acute lung injury, neutrophil apoptosis was delayed and macrophage activation released a large number of inflammatory factors (28–30), contributing to the apoptosis of alveolar epithelial cells and the exacerbation of acute lung injury. Surfactant protein A (SP-A) was in an interactive role with alveolar macrophages and participated in injury repair by modulating their function (33). Anti-inflammatory in ALI/ARDS in early-stage for blunt-injury in pediatrics were recommended in guidelines (28, 36). Monitoring of acute stress injury is also imperative in the progression and clinical management of ARDS.

Some limitations for this research were that adult and infant rabbits could be compared during different post-injury times. Then, mechanistic aspects were not fully explored in the present study and will need to be further investigated in future studies. Ciculating biomarkers for patients in blunt lung injury were in indeed. More high quality clinical studies are to be expected.

Therefore, monitoring serological SP-A can provide insights into the severity of lung injury in infants (31, 32). In cases of mild lung injury, immediately post-damage, even when infant rabbits exhibited minor damage changes on CT, alterations in injury and inflammation were evident. This underscores the necessity of closely monitoring the progression of the immediate inflammatory response in blunt lung trauma (36). Enhancing the anti-inflammatory content may effectively mitigate the progression of lung injury.

## Data Availability

The original contributions presented in the study are included in the article/Supplementary Materials, further inquiries can be directed to the corresponding author.
